# Can Digital Tools Be Used for Improving Immunization Programs?

**DOI:** 10.3389/fpubh.2016.00036

**Published:** 2016-03-08

**Authors:** Alberto E. Tozzi, Francesco Gesualdo, Angelo D’Ambrosio, Elisabetta Pandolfi, Eleonora Agricola, Pierluigi Lopalco

**Affiliations:** ^1^Unit of Telemedicine, IRCCS, Bambino Gesù Children’s Hospital, Rome, Italy; ^2^European Centre for Disease Prevention and Control, Stockholm, Sweden

**Keywords:** vaccination strategies, vaccination, immunization, immunization programs, registries, communication, vaccine confidence, information dissemination

## Abstract

In order to successfully control and eliminate vaccine-preventable infectious diseases, an appropriate vaccine coverage has to be achieved and maintained. This task requires a high level of effort as it may be compromised by a number of barriers. Public health agencies have issued specific recommendations to address these barriers and therefore improve immunization programs. In the present review, we characterize issues and challenges of immunization programs for which digital tools are a potential solution. In particular, we explore previously published research on the use of digital tools in the following vaccine-related areas: immunization registries, dose tracking, and decision support systems; vaccine-preventable diseases surveillance; surveillance of adverse events following immunizations; vaccine confidence monitoring; and delivery of information on vaccines to the public. Subsequently, we analyze the limits of the use of digital tools in such contexts and envision future possibilities and challenges.

## Introduction

Maintaining a high performance of an immunization program is one of the most challenging public health objectives. The history of immunization has shown several cases of success at a global level, including the eradication of smallpox and the control or elimination of several other vaccine-preventable diseases (1–3). Significant efforts and resources are constantly dedicated to supporting, maintaining, and improving immunization strategies, in order to achieve the goals set by national and international health agencies (2).

Several studies have been conducted to identify actions associated with an improvement of the vaccination coverage (4). In this regard, a collection of evidence-based recommendations has been issued by The Community Preventive Services Task Force (5).

Nevertheless, at a global level, a number of issues have become real threats for maintaining high immunization coverage, running an effective surveillance, and allowing immunization programs to timely react to new issues.

A large number of these issues could benefit from the adoption of digital tools (see Table 1). The objective of this review is to explore the role of potential digital solutions to problems of vaccination programs.

**Table 1:** **Potential uses of digital tools in immunization programs**.

Challenges	Addressed issues	Actions based on digital tools
Digitalization of immunization data	Need of data-driven activities in immunization programs	Implementation of immunization registries Integration of immunization registries with clinical information and other data
Improvement of logistics and dose tracking	Simplification of logistics in vaccine management, reduction of errors, and improvement of safety	Barcodes for dose tracking Integration of barcode scanning tech in EHRs
Decision support through appropriate algorithms for final users	Vaccination delay and vaccine hesitancy	Electronic decision support systems for health-care professionals Adoption of personal health records
Timely detection of epidemiologic signals	Delay in disease incidence reporting Low specificity of signals	Use of digital traces on the web for surveillance purposes Epidemic intelligence based on aggregation of different information sources Participatory surveillance
Improvement of vaccine safety evaluation	Underreporting and underrecognition of adverse events following immunizations	Integration and analysis of information on adverse events following immunizations obtained from EHRs and web signals
Timely assessment of the public’s vaccine confidence	Reduction of public confidence in vaccinations	Acquirement and interpretation of data on vaccine confidence from web sources and social networks
Effective delivery of vaccine information to the public	Reduction of public confidence in vaccinations Vaccine hesitancy Lack of effectiveness of common communication strategies for vaccine promotion	Addressing information gaps and misconceptions through analysis of digital traces left by users Integration of different digital tools for information delivery to the public

### Data Collection and Management

In most countries, especially in the developing world, the logistics of vaccination systems are paper-based, thus limiting timely update and information accessibility. Limited accessibility to vaccination information has a crucial impact on vaccination strategies, which cannot be timely and comprehensively informed by data. Monitoring vaccine safety and effectiveness can also be affected by lack of data accessibility. Moreover, in several countries, data on vaccinations are stored at the local level; therefore, citizens may have difficulties in accessing their vaccination record.

Regarding vaccination logistics, paper-based dose accountability has clear limits regarding safety and timely administration of doses according to schedules.

Digital solutions for data collection and management may streamline vaccination activities and provide important information to tailor immunization programs.

#### Immunization Registries

Immunization information systems (IISs) are confidential, population-based, computerized databases designed to record all immunization doses administered to a population, providing health operators with tools for maintaining a high vaccination coverage (6). IIS programs should provide solutions for (a) identification of at-risk individuals and groups; (b) management, storage, and integration of immunization data; (c) data protection; (d) facilitation in the engagement of families and individuals for timely vaccination receipt; (e) clinical decision support for health providers; and (f) framework for data sharing among health providers, at regional, national, and international levels (6).

Though several efforts have been made in some countries for adopting IIS, their use is far from being universal.

In Europe, only Denmark, Iceland, Malta, the Netherlands, and Norway have a national, fully implemented digital IIS, while six other countries have subnational IISs (7). Among the systems adopted by these countries, there is a high variability in methods, frequency of data acquisition, geographic coverage, data storage, and distribution (7, 8).

Initial efforts are being made to build digital registries at the international level, in order to facilitate global surveillance of vaccination programs and sharing of good practices. A system with such characteristics has been implemented by WHO and UNICEF: the Centralized Information System for Infectious Diseases (8).

Since 1998, Canada has had a network of regional IIS registries, which have been integrated in a national, intercommunicating network since the SARS pandemic in 2004 (9).

In the US, the CDC is working closely with electronic health record (EHR) providers to define the set of data and functionalities EHRs should have in order to be useful in the immunization setting, and to adopt a communication standard in order to allow consistent data intercommunication between health-care points and the CDC itself (10). Moreover, the American Academy of Pediatrics (AAP) is developing guidelines for tailoring immunization records to children, including parent refusal, information interchange with IISs, and clinical decision support functionalities (11).

#### Dose Tracking

Vaccine dose accountability may also benefit from digital solutions. The CDC vaccine tracking system (VTrckS) allows web-based ordering and tracking of publicly funded vaccines (12). The use of two-dimensional barcodes to store vaccine information (vaccine product identification, expiration date, and lot number) has been allowed by the FDA in 2011 (13, 14), is being experimented in a pilot study by the AAP and CDC (15, 16), and is already applied in Canada (17) and in Spain (13).

Integrating barcode scanning technology in EHRs has clear advantages in reducing errors and increasing safety (18, 19). In EHRs, an automatic link to vaccine information could also allow to easily tracking vaccine lots in case of adverse events following immunization (AEFI) (20).

#### Decision Support Systems

Clinical decision support systems help health professionals to correctly manage immunizations through the following functionalities: they suggest the appropriate immunization schedule for children based on birth date and vaccine history (21), automatically integrate changes in regulations (22, 23), proactively remind physicians of vaccinations for their patients (24), automatically recognize AEFIs (11), and suggest tailored immunizations in at-risk groups (25).

Personal health records (PHR) managed by patients and families can also improve adherence to immunization schedules through automatic notifications (26). A study performed on an adult population showed that the use of immunization PHRs correlates to a higher chance of receiving influenza immunization (27).

#### Developing Countries

Due to the lack of infrastructures and to high costs, adopting IISs in developing countries is a challenging task. Nevertheless, information systems enabling digital recording and transmission of immunization data are being implemented in Guatemala (28) and South Africa (29).

On the other hand, based on the observation that mobile phones are widely used in developing countries, mobile-based approaches may be promising in such contexts. For example, mobile technologies and advanced algorithms are being used to digitalize old paper-based immunization registries in low resource settings, e.g., Mozambique (30).

In Haiti, a cholera vaccination campaign has been carried out through house-by-house visits by operators equipped with wireless tablets. Children’s immunization status was assessed and recorded using a family-specific bar code; data were geolocalized and sent to a central system, which provided the program staff with a real-time map of vaccination coverage (31). A similar approach has been used in China, with a mobile app for facilitating immunization data recording, tracking unimmunized children, appointment booking (32) and in Thailand, with an app for recording data during antenatal and immunization visits (33).

### Tools for Vaccine-Preventable Diseases Surveillance

Traditionally, surveillance is defined as “the systematic collection, consolidation, analysis, and dissemination of data on specific diseases” (34). Classically, the main outputs of traditional surveillance systems have been indicators focused on individuals (34). More recently, surveillance activities have been aiming at rapidly capturing information about events that may represent a threat for public health and are referred to as event-based surveillance. Epidemiological surveillance is therefore extending from individual-based to event-based data (34, 35).

Traditional surveillance systems have a number of limitations:

- information is collected through health-care providers, not directly from individuals; therefore, traditional surveillance systems fail to catch signals from sick people who do not go to the doctor;
- traditional systems are based on case definitions, and therefore may miss emerging diseases with unexpected combinations of symptoms;
- there is a consistent time lag between signals of disease and production and dissemination of aggregated incidence figures.

In the context of event-based surveillance, information can be collected from news, reports, or other sources transmitted both through institutional and informal channels. Web-based data have been used to support public health in Canada since the 1990s, with the Global Public Health Intelligence Network (36), a service that automatically retrieves information about potential public health emergencies from news feed aggregators and distributes this information to public health agencies, including the WHO Global Outbreak Alert and Response Network (37).

Several studies explored the use and interpretation of spontaneous digital traces left by Internet users as a convenient and timely strategy to detect signals of trend variations in diseases and immunizations. The idea of using digital traces for syndromic surveillance was proposed by Eysenbach, who tracked demand for health information on the Internet using keyword-triggered ads for influenza (38). Subsequently, on the basis of the hypothesis that the interest of web users may correlate to disease incidence, a number of studies have focused on measuring the occurrence of specific health-related and disease-related search keywords.

Search volumes on web search engines may represent a surrogate of frequency of specific health events. A correlation between search volumes and disease trends has been shown (39). Search queries were useful to track dengue activity (40), and one study showed a correlation between search terms and laboratory confirmed cases of rotavirus infections (41). In 2008, a Google service (Google Flu Trends) has been developed to estimate and predict influenza activity by aggregating Google search query volumes (42). One study investigated the possibility of applying this approach to vaccinations, showing that search activity for HPV and H1N1 correlated to immunization coverage (43).

This “demand based” approach for surveillance has been subsequently integrated with the “supply based” approach, investigating communication contents and patterns in discussion groups, blogs, and microblogs (38), thus focusing on what users say, rather than on what users search for.

In particular, Twitter, a social network based on the sharing of short messages (up to 140 characters), which are available to the public without restrictions, has often been investigated as a source of information for infectious disease surveillance. Twitter posts are rich in data, allowing to follow disease trends both temporally and geographically. Many studies have explored whether monitoring information flow and networks on Twitter could help following the emergence of health conditions, their evolution, and the public’s interest around them. Influenza surveillance has been one of the main topics in Twitter research (44), with international (45) and local scale studies (46, 47). This approach has been recently used to study the incidence trends of other infectious diseases, namely, pertussis (47), dengue (48), and cholera (49).

On the other hand, vaccine-preventable disease surveillance may benefit from systems based on an active input of information by users. Such methods, grouped under the definition of “participatory surveillance,” are based on platforms (both web- and smartphone-based) that allow users to directly provide information about their health status. Typical examples of this kind of surveillance are platforms dedicated to crowdsourced influenza surveillance (e.g., Flu Near You or Influnet) (50–52), providing a powerful and precise tool for epidemic assessment.

Complex biosurveillance systems aggregate data from a variety sources: news sites, social media, crowdsourcing platforms, official resources (e.g., WHO), audio, and video sources. Information acquired by such platforms may provide geographical details both at the local and at the international level (53). One of the best examples of Internet biosurveillance systems is represented by Healthmap, which provides information about emerging and re-emerging public health threats by aggregating information from various structured and non-structured data sources (54).

GeoChat (55) is another example of an open source platform that can enable the easy deployment of crowdsourced interactive mapping applications for surveillance with web forms/e-mail, short message service (SMS), and Twitter support. This application has been used in Cambodia for disease reporting and outbreak alerts (56).

Epidemic intelligence activities can also integrate traditional surveillance systems for the detection of vaccine failure and lack of effectiveness. Detection of outbreak clusters in vaccinated populations may trigger a signal of potential vaccine failure. Such signals must be verified and properly assessed using “traditional” epidemiological techniques in order to get estimates of vaccine effectiveness and either confirm or reject the signal (57).

### Tools for Surveillance of Adverse Events Following Immunizations

Assessment of vaccine safety is a priority for public health and may have a powerful impact on the success of an immunization program. AEFI may be studied before marketing authorization, in the context of phase I-III clinical trials, which, though, may not have a sufficient magnitude for adequately detecting rare AEFIs. Subsequently to licensure, AEFIs are monitored through *ad hoc*, formal studies, or, more frequently, through passive surveillance by health-care workers. Limits of such systems are under-reporting and biased reporting (8).

Innovative ways to monitor AEFIs and capture signals of vaccine safety have been explored extensively (58).

In particular, large databases of EHRs have been used to set up systems able to capture signals in a semi-automated way. The US Vaccine Safety Datalink (VSD) is the oldest of such infrastructures, established by the CDC in 1990 for population-based, post-marketing monitoring of vaccine safety (59). The VSD project includes a population of nearly 9 million individuals, including 2.1 million children, attending various participating organizations. In addition, the FDA has recently established the Post-Licensure Rapid Immunization Safety Monitoring (PRISM) system, built on the VSD model, linking information from different health insurance databases (60). Such systems are extremely efficient in addressing specific questions related to vaccine safety in a timely manner. On the other hand, they are not specifically designed to detect safety signals.

Clusters of mass psychogenic illness following pandemic influenza vaccination were detected and investigated in Taiwan in 2009 using a combination of institutional data sources (the passive AEFI surveillance system and the web-based Emergency Medical Management System) (61). Association between narcolepsy and H1N1 pandemic vaccine Pandemrix in Finland was initially flagged up by an informal digital network of neurologists and was afterward confirmed by epidemiological studies (62). The same signal would have been hardly detected through the routine AEFI passive reporting system, being an unexpected and rare event.

Surveillance of AEFIs through a participatory approach has been a matter of debate. In many AEFI surveillance systems, patients do report health events. Post-marketing surveillance of AEFI has been implemented through mobile devices (63) and SMS (64).

### Monitoring Vaccine Confidence

The media resonate with uncontrolled, scaring information on vaccine safety, and the general public is exposed to conflicting information on the balance between risks and benefits of immunizations. As a matter of fact, vaccine hesitancy, due to lack of confidence in vaccinations, may have a crucial impact on vaccine uptake (65, 66).

Vaccine confidence is traditionally measured through classic surveys or interviews (67, 68).

As information regarding vaccinations is largely acquired on the Internet, web data mining may enlighten various aspects of vaccine confidence. Parents seeking vaccine information on the Internet, compared to those using other information sources, are less likely to agree with accepted principles of vaccine science and less likely to recognize the benefits of vaccinations (69). Side effects, ingredients, and immunization policies are the most searched and discussed topics on the web (70–72).

Web monitoring of vaccine confidence may allow to timely intercept negative trends and, therefore, to set up immediate actions. This objective may be achieved by collection and interpretation of heterogeneous data on vaccines derived from various web sources (73), including social networks (74).

### Delivering Information on Immunizations to the Public

Vaccine information campaigns are mostly based on paper material and delivered through traditional media channels. This approach has several limitations. First, a large time lag may exist between the detection of information needs and information delivery. Second, the tailoring potential of this approach is limited, as information campaigns usually target the general population.

Digital tools and new media can be exploited as means for accurately identifying information needs and effectively delivering vaccination campaigns. A number of studies have investigated the presence of vaccine information on the web, often starting from an analysis of Google search results obtained through the use of specific keywords (72, 75–78). This approach can be useful for addressing information trends and gaps on available web pages.

Information on vaccinations published on the web, as well as most common information gaps and misconceptions, have often been assessed through an analysis of vaccine-related websites. A number of studies have analyzed both websites promoting vaccinations and anti-vaccination websites (79–81). To this regard, the World Health Organization conducts the Vaccine Safety Network, an initiative aimed at crediting web sites meeting certain quality criteria, which are periodically reviewed (82).

For a complete assessment of information trends on vaccinations, social networks cannot be neglected, as they are a fertile medium for anti-vaccine sentiments (83). Blogs commonly support variable positions on vaccinations (84); nevertheless, negative messages may have a greater influence on the decision to vaccinate, compared to pro-vaccine messages (85). Twitter has been studied as a source of information on vaccines (86), with pro-vaccine contents being more prevalent on Twitter compared to other social media (87). Moreover, the use of social media and, specifically, of Facebook, seems promising for the implementation of communication strategies targeting adolescents, who are particularly engaged in this kind of media. In this regard, a number of studies investigated patients’ enrollment and HPV vaccination promotion on Facebook (88–91).

Recently, some attempts have been conducted to pilot tailored communication toward vaccine-hesitant parents (92, 93). A successful tailored education on HPV vaccine has been experienced by Chinese health professionals who collaborated with popular websites for women to gear up vaccination uptake (94).

A large part of the literature on digital tools aimed at improving immunization uptake focuses on electronic reminders. Reminders can be included in more complex interventions to improve immunization coverage (95). Several authors have documented the impact of text messaging and computerized reminders in increasing adherence to schedules, especially if integrated with educational interventions (27, 96–99). On the other hand, the evidence supporting these interventions, as for others digital tools for public health, seems insufficient (24).

## Comments and Discussion

Only small progresses have been made to integrate digital tools into immunization programs. Moreover, research aimed at assessing the effectiveness of digital technologies as potential responses to problems of immunization programs is scarce.

Electronic data management is not fully applied to traditional components of immunization programs yet. Electronic data on vaccinations should be integrated at the international level, in order to facilitate cross collaboration and coordination among health services and research institutes. Moreover, easy access to large amounts of immunization records may give a robust contribution to vaccine research, facilitating the design of timely and inexpensive epidemiological studies to assess vaccine efficacy and safety (9).

With regard to surveillance, crowdsourcing, mobile phones, personal computing devices, and geolocalization of information promise to become stable pillars of public health strategies. Combining these resources with other tools, such as SMS and social networks, may generate innovative instruments to support surveillance of health events and other public health activities (56). This observation extends to monitoring vaccine coverage and AEFI occurrence, where social media can enhance traditional information sources.

The analysis of search queries and social media information is a powerful approach, although it may suffer from information biases, as interest in specific diseases may be amplified by media attention, independently from the disease incidence (100, 101). Nevertheless, refined algorithms and the use of text mining techniques for sense disambiguation, topic filtering, and mood analysis (102, 103) are allowing to isolate actual signals of disease from information noise with increasing precision. The use of natural language processing techniques and of algorithms allowing an automatic or semi-automatic classification of contents may also allow to overcome the manual evaluation of contents, which is an expensive and energy-consuming task. One step toward a more accurate surveillance may be represented by participatory surveillance. A recent review showed a large potential benefit of participatory surveillance, although specificity may be limited and participating bias can affect its performance (104).

Parallel to surveillance of medical events, the use of digital tools for monitoring vaccine confidence and information needs may greatly enhance the performance of immunization programs. Vaccine hesitancy and vaccine opposition are increasingly worrying phenomena. The detection of a drop in vaccine confidence may anticipate a more severe event, such as a decrease in vaccine coverage, allowing the immunization program to react. Monitoring information needs may allow to identify issues which are potentially linked with vaccine hesitancy or opposition. Moreover, data gathering and analysis of information found on the web has the advantage to shortcut traditional channels and to allow continuous monitoring and interpretation of data (73). An interesting approach for investigating information needs may be represented by the analysis of search behaviors through the study of search terms and queries, which are commonly used by Internet users (105, 106), using specific platforms such as Google Adwords.

On the other hand, monitoring vaccine confidence and information needs on the Internet does not allow to consider segments of the public that do not have access to the Internet or that do not leave digital traces on the web. Therefore, information obtained from the Internet is not sufficient to describe the entire population and should be used to integrate other traditional sources of information. Nevertheless, web-derived information may allow to finely profile specific populations of Internet users, which may become the target of tailored immunization campaigns delivered through multiple web channels.

Business marketing strategies are based on rules for translating information delivery into monetary return of investments. The same approach may be used to rapidly spread vaccine information to different segments of the public to maintain vaccine confidence and increase immunization coverage. Information strategies should exploit more comprehensively the speed and pervasiveness of digital tools. The web community will likely become increasingly demanding, switching from a passive acceptance of static, limited contents to an active request of detailed information. Vaccine information delivery should therefore move from classical, static web sites to real interactions with the users, especially through the use of social networks. The future scenarios of immunization policies will possibly be characterized by a direct participation of the public in designing appropriate information strategies and even efficacious approaches to maintaining immunization programs.

All the potentials hitherto described can be greatly enhanced when the tool used for interaction is a mobile phone. Indeed, people who access the Internet through a smartphone rather than a computer are more likely to interact with the technology than simply consuming information (107). Apps dedicated to vaccination might be highly impacted in this population group. On the other hand, one of the issues in using electronic reminders based on cellular phone may be that the global penetration of these devices is not universal (108).

At present, a number of barriers limit the adoption of digital tools in immunization programs. First, ability to use digital tools may be hampered by cultural background and infrastructure availability. This implies an urgent need for educational activities aimed at empowering health professionals and patients for the use of such tools. Institutional stakeholders should drive political decisions toward the use of digital tools both in research and clinical activities. Second, the adoption of digital tools requires a relatively large, initial investment in human and financial resources. This may represent a limit, in particular, in developing countries or in countries suffering from financial crisis. Nevertheless, the return of such investments is high in terms of increased quality of immunization programs and subsequent cost savings. Third, the use of Internet for managing health data is subject to security and privacy issues. Strategies to maintain anonymity and preserve confidentiality are difficult to implement. Moreover, relying on proprietary resources, such as those offered by Google, may be problematic, since algorithms used by this company are not explicit (109).

Research studies assessing the impact of digital tools in immunization programs are still rare and do not follow the rapid pace of development of technology and digital tools. This discrepancy is common to other domains and should be rapidly filled up to improve immunization programs.

In conclusion, despite digital tools may greatly enhance the effectiveness of immunization programs, only few examples of implementation are available at present. The use of digital tools can favor the intersection of three crucial dimensions of immunization programs: immunization registries, surveillance of vaccine-preventable diseases, and surveillance of AEFI. The fourth dimension, represented by monitoring confidence in immunization programs, can be easily integrated through the use of digital instruments, which would allow implementation of data-driven vaccine information strategies. An expanded use of digital tools is expected to ultimately increase immunization coverage, reduce vaccine-preventable disease incidence, reduce AEFIs, and increase the active participation of the public to immunization strategies through informed decisions.

## Author Contributions

AET, FG and AED conceived the project and wrote the article. EP and EA performed the literature review. PL critically revised the article and gave the final approval for publication.

## Conflict of Interest Statement

The authors declare that the research was conducted in the absence of any commercial or financial relationships that could be construed as a potential conflict of interest. The Reviewer SW-C and handling Editor PA declared their shared affiliation, and the handling Editor states that the process nevertheless met the standards of a fair and objective review.
